# Transdermal delivery of fluvastatin sodium *via* tailored spanlastic nanovesicles: mitigated Freund's adjuvant-induced rheumatoid arthritis in rats through suppressing p38 MAPK signaling pathway

**DOI:** 10.1080/10717544.2019.1686087

**Published:** 2019-11-18

**Authors:** Shahira F. El Menshawe, Mohamed M. Nafady, Heba M. Aboud, Rasha M. Kharshoum, Asmaa Mohammed M. Hussein Elkelawy, Doaa S. Hamad

**Affiliations:** a Department of Pharmaceutics and Industrial Pharmacy, Faculty of Pharmacy, Beni-Suef University, Beni-Suef, Egypt;; b Department of Pharmaceutics and Clinical Pharmacy, Faculty of Pharmacy, Nahda University, Beni-Suef, Egypt;; c Department of Pharmacology, Faculty of Medicine, Beni-Suef University, Beni-Suef, Egypt

**Keywords:** Fluvastatin sodium, spanlastic nanovesicles, transdermal delivery, rheumatoid arthritis, p38 MAPK

## Abstract

The current study aimed to encapsulate fluvastatin sodium (FVS), a member of the statins family possessing pleiotropic effects in rheumatoid arthritis (RA), into spanlastic nanovesicles (SNVs) for transdermal delivery. This novel delivery could surmount FVS associated oral encumbrances such as apparent first-pass effect, poor bioavailability and short elimination half-life, hence, accomplishing platform for management of RA. To consummate this objective, FVS-loaded SNVs were elaborated by thin film hydration method, utilizing either Span 60 or Span 80, together with Tween 80 or Brij 35 as an edge activator according to full factorial design (2^4^). Applying Design-Expert^®^ software, the influence of formulation variables on SNVs physicochemical properties and the optimized formulation selection were explored. Additionally, the pharmacokinetic studies were scrutinized in rats. Furthermore, in Freund's adjuvant-induced arthritis, rheumatoid markers, TNF-α, IL-10, p38 MAPK, and antioxidant parameters were measured. The optimum SNVs were nano-scaled spherical vesicles (201.54 ± 9.16 nm), having reasonable entrapment efficiency (71.28 ± 2.05%), appropriate release over 8 h (89.45 ± 3.64%) and adequate permeation characteristics across the skin (402.55 ± 27.48 µg/cm^2^). The pharmacokinetic study disclosed ameliorated bioavailability of the optimum SNVs gel by 2.79- and 4.59-fold as compared to the oral solution as well as the traditional gel, respectively. Moreover, it elicited a significant suppression of p38 MAPK expression and also significant improvement of all other measured biomarkers. Concisely, the foregoing findings proposed that SNVs can be auspicious for augmenting FVS transdermal delivery for management of RA.

## Introduction

Rheumatoid arthritis (RA) is a chronic inflammatory, autoimmune ailment which impinges joints beside other tissues. Articular demolition, inflammatory synovitis as well as coordinated peripheral polyarthritis are the hallmarks of RA. It is depicted by pain, stiffness, and swelling of joints that precede bone and joint eradication resulting in fast incidence of significantly clinical functional feebleness. RA represents a frugal burden for the society and health disciplines (Taal et al., 2006). There are various therapeutic choices for RA including non-steroidal anti-inflammatory drugs, analgesics, corticosteroids, disease-modifying anti-rheumatic drugs in addition to biologic therapies. Though, current therapies elucidated their efficacy in treatment of RA, they do not substantially amend the underlying mechanisms responsible for RA progression (Li et al., 2017). Moreover, these therapies elicit severe side effects following few intervals of treatment and often accompanied with inadequate results. Thus, novel curative strategies that are safer on long-term treatment of RA are warranted (Paraskevas, 2008).

Due to their anti-inflammatory and immunomodulatory characteristics, statins are proposed as appropriate candidates for treatment of RA (Arnaud & Mach, 2006; Soulaidopoulos et al., 2018) where thorough *in vitro* data, experimental and clinical studies have robustly pointed out statins to exert pleiotropic effects in RA (Jury & Ehrenstein, 2005; McCarey et al., 2005).

Fluvastatin sodium (FVS) is a member of the stain family that is estimated as the first line choice for treatment of hyperlipidemia (Yano et al., 2009). FVS inhibits HMG-CoA reductase responsible for cholesterol synthesis in the liver and consequently, suppresses the plasma concentration of low-density lipoproteins (LDLs). Elevated LDLs plasma levels increase the risk of coronary artery disease, atherosclerosis, and plagues development with life-intimidating outcomes (Kah et al., 2012). Interestingly, experimental findings have proved the valuable role of FVS against RA where it abolishes endothelial dysfunction and abates oxidative stress in rat model of arthritis (Haruna et al., 2007). Furthermore, FVS may be beneficial in killing inflammatory cells in RA as it has been reported to induce apoptosis (Fukumoto et al., 2001). It is worth mentioning that one of the most important signal transduction systems in mediating cartilage injury observed in RA is mitogen activated protein kinase (MAPK) signaling pathway (Zhang et al., 2015). Activation of the MAPK pathway is related to inflammatory responses to induce the expression of various inflammatory genes being a risk factor for the persistence and severity of RA (Haruna et al., 2007). It includes p38 MAPK, extracellular signal-regulated kinase (ERK), and c-Jun N-terminal kinase (JNK) (Ahmed et al., 2017). p38 MAPK activation was feature observed in the synovial lining layer and in synovial endothelial cells (Waaler, 2007). However, effects of statins on MAPK cascades in cells from RA synovium are not well known as their inhibitory effects on MAPKs have been studied in vascular smooth muscle cells and endothelial cells not in RA (Petersen et al., 2010). Hence, we attempted to investigate the effect of FVS on p38 MAPK expression in RA.

Unfortunately, FVS encounters low oral bioavailability (24%) and short plasma half-life of 1–3 h as a result of being extensively metabolized by cytochrome P3A in the gut wall and liver (Scripture & Pieper, 2001). Regarding FVS utilization for long-term treatment, it is necessary to ameliorate its bioavailability in addition to attain a sustained release behavior to decrease both the dose and the frequency, thus enhancing patient compliance. Therefore, administration by an alternative route is a key requirement to tackle such drawbacks. The current study converged on investigating transdermal route for FVS administration. Recently, Kaur & Ajitha (2019) declared ameliorated anti-osteoporotic activity in rats of transdermally applied FVS nanoemulsion gel.

The transdermal route is commonly deemed as a patient amiable option as it avoids GIT adverse effects, variable pH conditions along GIT and first-pass metabolism that often entail oral drug administration. Also, it provides a constant and sustained drug effect in the body and its impact can be simply reversed (Al-Kassas et al., 2016). Moreover, transdermal drug delivery precludes fluctuations in drug plasma level which eventually diminishes both adverse reactions and incomplete drug therapy. Nevertheless, the barrier feature of the stratum corneum (SC) is counted the utmost impediment for transdermal delivery. Several approaches have been pursued to boost transdermal drug delivery including the implementation of nanovesicular carriers (Mahmoud et al., 2017).

Spanlastic nanovesicles (SNVs) are nonionic surfactant-structured vesicles characterized by high elasticity and deformability in which surfactant macromolecules are organized as bilayer membrane utterly enclosing solute aqueous solution (Fahmy et al., 2019). They are flexible-walled Sorbitan tailored vesicles fabricated by modification of the classical niosomes *via* edge activator (EA) amalgamation during the formulation process (Abdelrahman et al., 2017). Incorporation of EAs imparts elasticity by destabilization of the vesicles and fluidization of their bilayers by lowering the interfacial tension. SNVs have the aptitude to breach the SC fence and to invade intensely through the target dermal tissues by squeezing themselves throughout SC intercellular domains as a result of their ultra-deformability. This novel nanocarrier can boost transdermal drug penetration (Abbas & Kamel, 2019).

The utilization of SNVs for the enhancement of duodenum-triggered (Tayel et al., 2015), ocular (Kakkar & Kaur, 2011), trans-tympanic (Al-Mahallawi et al., 2017) as well as trans-ungual drug delivery (Elsherif et al., 2017) was previously reported. Only, the present study investigates the potential of these elastic vesicles for FVS transdermal delivery for management of RA.

The aim of this investigation was to deliver FVS transdermally *via* ultra-flexible surfactant-based nanovesicles dispersed into gel in order to enhance its systemic absorption and to modulate RA. A combination of different Spans (Span 80 or Span 60) with diverse EAs (Brij 35 or Tween 80) was employed for development of SNVs. Physicochemical appraisal of the developed SNVs was executed to scrutinize their convenience for transdermal delivery. Also, pharmacokinetic studies in rats were carried out comparing the pharmacokinetic parameters of FVS after oral and transdermal administrations. Furthermore, an *in vivo* study was constructed to test the prepared formulation in rat model of Freund's adjuvant-induced arthritis.

## Materials and methods

### Materials

Fluvastatin sodium was kindly donated by Eipico (Cairo, Egypt), Span 60 (sorbitan monostearate), Span 80 (sorbitan monooleate), Tween 80 (polyoxyethylene sorbitan monooleate), Brij 35 (polyoxyethylene lauryl ether), acetonitrile (HPLC grade), methanol (HPLC grade), diethyl ether (HPLC grade), complete Freund's adjuvant (CFA), and methotrexate were acquired from Sigma-Aldrich (St. Louis, MO). Dialysis bags with a molecular weight cut off of 12,000 Da were obtained from SERVA Electrophoresis GmbH (Heidelberg, Germany). Serum rheumatoid factor (RF), cartilage oligomeric matrix protein (COMP), and antinuclear antibody (ANA) ELISA kits were purchased from CliniLab Company (Cairo, Egypt). Serum tumor necrosis factor-alpha (TNF-α) ELISA kit was obtained from Glory Science company (St. Del Rio, TX). Serum interleukin-10 (IL-10) ELISA kit was obtained from Cusabio Biotech Co. (Wuhan, China). Serum malondialdehyde (MDA) and glutathione (GSH) reduced colorimetric kits were purchased from Biodiagnostic Company (Giza, Egypt). Acrylamide Kit (SDS-PAGE) (for sodium dodecyl sulfate polyacrylamide gel electrophoresis) was provided by Bio-Rad Laboratories (TNC, Hercules, CA) catalog no. 161-0181. All other ingredients used were of analytical grade.

### Preparation of FVS-loaded SNVs

FVS-loaded SNVs were prepared according to thin film hydration technique (Al-Mahallawi et al., 2017). In this technique, Span 60 or Span 80 was weighed into a round-bottom flask and dissolved in chloroform, and then by using a rotary evaporator (Heidolph Laborota 4000 Series, Heizbad, Germany) at 90 rpm, this organic solvent was evaporated at 60 °C under vacuum, resulting in formation of a thin film on the flask wall. Subsequently, the resultant film was hydrated with 10 ml phosphate-buffered saline (PBS) pH 7.4 containing 10 mg FVS together with the EA, by rotation of the flask within a water bath at 60 °C for 30 min under normal pressure. Certain SNVs formulations, according to the experimental design indicated in Table 1, were bath-sonicated (Tianjin Automatic Science Instrument Ltd, Nanyang, China) for 5 min. The vesicular dispersions were kept overnight at 4 °C to mature and then used for further investigation.

**Table 1.** The 2^4^ full factorial design and the observed responses of FVS-loaded SNVs.

Formulation	Independent variables				Dependent variables				PDI
	A: Span type	B: EA type	C: EA concentration (% w/w)^a^	D: Sonication time (min)	Y_1_: EE%	Y_2_: Vesicle size (nm)	Y_3_: Q_8h_ (%)	Y_4_: Q_24_ (µg/cm^2^)	
S1	Span 60	Tween 80	10	0	65.70 ± 2.55	692.57 ± 17.03	67.23 ± 11.75	229.44 ± 20.45	0.45
S2	Span 60	Tween 80	20	0	80.07 ± 3.10	388.40 ± 18.86	73.36 ± 11.50	284.45 ± 15.32	0.34
S3	Span 60	Brij 35	10	0	61.13 ± 1.42	569.40 ± 17.62	70.88 ± 9.00	255.51 ± 19.50	0.48
S4	Span 60	Brij 35	20	0	78.70 ± 1.21	322.07 ± 19.33	77.23 ± 5.25	292.12 ± 21.03	0.50
S5	Span 80	Tween 80	10	0	57.65 ± 2.09	275.07 ± 18.06	79.23 ± 3.75	313.96 ± 22.50	0.37
S6	Span 80	Tween 80	20	0	77.13 ± 2.26	220.53 ± 21.34	87.25 ± 3.25	349.42 ± 21.10	0.27
S7	Span 80	Brij 35	10	0	54.40 ± 1.49	250.57 ± 16.87	83.24 ± 3.25	324.42 ± 25.50	0.34
S8	Span 80	Brij 35	20	0	73.13 ± 2.30	215.00 ± 20.00	90.92 ± 4.00	392.67 ± 23.02	0.34
S9	Span 60	Tween 80	10	5	43.17 ± 1.77	576.67 ± 21.73	69.52 ± 2.50	265.99 ± 24.32	0.49
S10	Span 60	Tween 80	20	5	58.40 ± 2.04	329.33 ± 24.01	73.80 ± 3.75	290.06 ± 20.45	0.38
S11	Span 60	Brij 35	10	5	42.53 ± 2.45	380.00 ± 21.79	71.24 ± 3.75	277.54 ± 22.52	0.37
S12	Span 60	Brij 35	20	5	50.17 ± 2.02	280.20 ± 17.79	80.45 ± 2.50	308.77 ± 21.21	0.32
S13	Span 80	Tween 80	10	5	40.50 ± 2.50	264.17 ± 11.77	81.47 ± 3.51	325.06 ± 25.02	0.46
S14	Span 80	Tween 80	20	5	46.77 ± 3.03	170.00 ± 10.00	88.89 ± 3.25	402.48 ± 25.50	0.33
S15	Span 80	Brij 35	10	5	34.27 ± 2.15	230.53 ± 10.45	84.43 ± 4.50	338.50 ± 21.50	0.45
S16	Span 80	Brij 35	20	5	43.33 ± 3.06	167.83 ± 11.73	92.08 ± 3.00	460.59 ± 20.01	0.35

EA: edge activator; EE%: entrapment efficiency percent; Q_8h_: cumulative release after 8 h (%); Q_24_: cumulative amount permeated/unit area in 24 h; PDI: polydispersity index.

Data are mean values (*n* = 3) ± SD.

a (% w/w) of the EA with respect to Span/EA total weight.

### Statistical design of the study

A 2^4^ full factorial experimental design was adopted to explore the impact of the applied variables on the characteristics of SNVs dispersions employing Design-Expert^®^ software (Version 10, Stat-Ease Inc., Minneapolis, MN). The independent variables were Span type (A), EA type (B), EA concentration (C), and sonication time (D). The investigated responses were EE% (Y_1_), vesicle size (Y_2_), cumulative % FVS released from SNVs after 8 h (Q_8h_) (Y_3_), and cumulative amount of FVS permeated/cm^2^ after 24 h (Q_24_) (Y_4_) as dependent variables, Table S1 (supplemental file).

### *In vitro* characterization of FVS-loaded SNVs

#### Determination of FVS entrapment efficiency percent

Entrapment efficiency percent (EE%) analysis was carried out, in triplicate, by cooling centrifugation method (Elkomy et al., 2016). Briefly, FVS-loaded SNVs dispersions were centrifuged at 14,000 rpm for 2 h at 4 °C (Sigma Laborzentrifugen, Osterode am Harz, Germany). The precipitated FVS-loaded SNVs were washed with PBS pH 7.4 twice then, the clear supernatant was separated each time from SNVs and filtered with a 0.45 nm nylon syringe filter then assayed for free non-entrapped drug at 233 nm using UV spectrophotometer (Shimadzu UV-1800, Tokyo, Japan). The EE% of FVS was calculated using the following equation:

(1) $$\mathrm{EE}\%=\frac{\text{total drug concentration}-\text{free drug concentration}}{\text{total drug concentration}}\;\times \;100$$

#### Determination of SNVs' size

The average vesicle size and polydispersity index (PDI) of the prepared FVS-loaded SNVs were assessed using Zetasizer Nano 7.11 (Malvern Instruments, Malvern, UK). Dilution of 0.1 ml of each dispersion was performed with distilled water to 10 ml before the measurements to abolish the multi-scattering phenomena. All measurements were performed in triplicate at 25 °C and an angle of 90° to the incident beam (Aboud et al., 2018) and the mean values ± SD obtained were reported.

### *In vitro* drug release studies

The *in vitro* release profiles of FVS-loaded SNVs dispersions were conducted and compared to free FVS solution in a USP I dissolution apparatus (Erweka DT-720, Erweka GmbH, Heusenstamm, Germany) adopting dialysis bag diffusion method (Salem et al., 2015). Briefly, SNVs dispersion of different formulations (equivalent to 3 mg of FVS) was introduced into glass cylinders (6 cm length and 2.5 cm internal diameter) firmly covered from one end with the dialysis membrane with molecular weight cut off of 12,000 Da which was soaked in the receptor milieu overnight. The loaded cylinders were anchored at the shafts of the USP dissolution tester apparatus (Abdelrahman et al., 2017). The release milieu was 250 ml Sorensen's phosphate buffer pH 5.5 to ensure sink condition (El-Helw & Fahmy, 2015). Rotation speed was adjusted to be 100 rpm with temperature set to 32 ± 0.5 °C over the release study. Aliquots (3 ml) were withdrawn from the release milieu after 0.5, 1, 2, 3, 4, 6, and 8 h and were replenished with an equal volume of fresh milieu to ensure constant volume. FVS content was spectrophotometrically assayed at *λ*_max_ 233 nm after filtration of the samples through 0.45 μm membrane filter. The release study was performed in triplicate for all formulations and the results were expressed as the mean values ± SD. The cumulative percent of FVS-loaded SNVs released was plotted against time. For analysis of the release kinetics of FVS-loaded SNVs, the procured data were fitted into zero-order, first-order, Higuchi, Korsmeyer–Peppas, and Hixson–Crowell models. Picking of the adequate mathematical model was relied on the magnitude of the coefficients of determination (*R*^2^).

### *Ex vivo* permeability study

Wistar rats (6–8 weeks old, 100–125 g) were euthanized by decapitation under anesthesia and the dorsal skin of each rat was excised. Hairs on the skin of animals were stripped by shaving, SC tissues were surgically separated and the dermis side was rubbed with isopropyl alcohol to detach residual adhering fat and the skin was washed with PBS pH 7.4. The skin samples were mounted over the diffusion cells in such a way that the SC side faced the donor compartment while the dermis faced the receptor compartment of vertical Franz diffusion cell (Ahad et al., 2012; Mahmoud et al., 2017). Franz diffusion cell of 5 cm^2^ surface area was used for *ex vivo* diffusion studies. The receiver chamber contained 100 ml of PBS pH 7.4 as diffusion milieu which was maintained at 37 ± 0.5 °C and stirred at 100 rpm. The experiment was carried out, in triplicate, under non-occlusive state. Different volumes of SNVs dispersion holding constant quantities of FVS (3 mg) were added in the donor compartment of Franz diffusion cell. Samples from the receptor compartment were withdrawn at predetermined time intervals (1, 2, 4, 6, 8, 12, and 24 h) then the receptor chamber was compensated with equal volumes of fresh milieu. The withdrawn samples were filtered through 0.45 µm membrane filter and finally measured at 233 nm using spectrophotometer and analyzed.

For each formulation, the cumulative amount of drug permeated per unit area (μg/cm^2^) was plotted versus time (h). The permeation parameters involving Q_24_ in μg/cm^2^, lag time in min, permeability coefficient (Kp) in cm/h, drug flux (Jss) in μg/cm^2^ h were computed for each formulation and for the control FVS solution. Additionally, the enhancement index (EI) was calculated applying the following equation (El-Nabarawi et al., 2016):

(2) $$\mathrm{EI}=\;\frac{\text{Kp of SNVs}}{\text{Kp of control solution}}$$

### Optimization of FVS-loaded SNVs

Design-Expert^®^ software was adopted to choose the optimized SNVs dispersion by employing the desirability function. The optimization process was planned to select a formulation with the greatest EE%, Q_8h_, and Q_24_ as well as the smallest vesicle size, Table S1 (supplemental file). The solution with desirability value near to one was picked. For emphasizing the model validity, the selected formulation was prepared, evaluated and eventually, compared with the predicted responses.

### Transmission electron microscopy (TEM)

Visualization of the morphological appearance of the optimal SNVs was performed adopting transmission electron microscope (JEM-1400, Jeol, Tokyo, Japan) operating at 80 kV. One drop of the diluted vesicular dispersion was deposited on the surface of a carbon-coated copper grid, negatively stained with 2% phosphotungstic acid then allowed to dry at room temperature for 10 min before investigation (Mahmoud et al., 2017).

### Differential scanning calorimetry (DSC)

The thermal properties of pure FVS, components and the optimal FVS-loaded SNVs were investigated using DSC (DSC 50 Shimadzu, Kyoto, Japan). Five mg-samples were crimped in a standard aluminum pan and heated from 25 to 300 °C at a constant heating rate of 5 °C/min under constant purging of argon at 20 ml/min (El-Nabarawi et al., 2016).

### Physical stability study of SNVs

The optimized SNVs formulation was subjected for stability investigation through storing in a glass vial for 3 months at 4 °C. Samples from the optimized formulation were withdrawn after preparation, then after predetermined time intervals of 30, 60, and 90 days of storage. The withdrawn samples were characterized for EE%, particle size, and size distribution and measurements were repeated three times (Mahmoud et al., 2017).

### Skin irritation studies

#### Animals

The optimized SNVs formulation was converted into gel by incorporating 1% (w/w) carbopol 971P for transdermal application. The safety of transdermal FVS-loaded SNVs gel treatment was tested in a group of 12 female albino Wistar rats weighing 200–250 g obtained from animal facility of Nahda University (Beni-Suef, Egypt). All animals were nourished with normal chow and tap water *ad libitum*. The animals were grouped randomly (normal control and gel treated) and housed in polyacrylic cages (six animals per cage) and kept under standard laboratory circumstances. Rats were set for applying the gel on rats' dorsal side for two weeks after shaving the hairs. The incidence of adverse effects as skin irritation and erythema was observed at the gel application site of rats over the treatment period for evaluation of treatment safety. By the end of the study, rats were killed by cervical decapitation under anesthesia and the gel application area as well as non-application area (served as a control) of the same animal were removed, rinsed with normal saline and preserved in 10% neutral buffered formalin for histopathological investigation. This study was approved by our institutional animal ethics committee of Beni-Suef University.

### Histological examination

Skin specimens were collected randomly from three rats of each group. Tissues were perfused with normal saline and preserved in 10% neutral phosphate buffered formalin and embedded in paraffin. The prepared section was then deparaffinized using xylol and subsequently hydrated in ethyl alcohol. After dehydration, paraffin-embedded tissue sections (5 μm) were stained with hematoxylin and eosin (H&E) according to the method of Bancroft & Gamble (2008). Sections were photographed using a digital camera attached to a light microscope.

### Pharmacokinetic studies

#### FVS administration to rats

The protocol of this study was reviewed and approved by our institutional animal ethics committee of Beni-Suef University (protocol approval no. REC-A-PhBSU-19013) and all procedures for agent administration, blood and tissue collection were in accordance with the 8^th^ edition of the Guide for the Care and Use of Laboratory Animals published in 2011 by the United States National Academy of Sciences. Eighteen female mature Wistar rats weighing 200–250 g, obtained from animal facility of Nahda University (Beni-Suef, Egypt), were used for *in vivo* study to scrutinize the variations in pharmacokinetic parameters of FVS after administration. All rats were randomly divided into three groups, six rats each. FVS was administered in a dose of 5 mg/kg (Haruna et al., 2007). The first group administrated an oral FVS solution (1 mg/ml) in purified water, the second and third groups were administrated FVS transdermal dose of the optimized SNVs gel and traditional gel, respectively. Animals were allowed free access to food and water, until the night prior to dosing and were fasted for 10 h. The oral solution was administered through oral gavage. The rats to be used for application of SNVs and traditional gels were shaved and then the site cleaned with water before application of the gel. The gel (1 g) was applied on the dorsal surface of each test animal over a surface area of 4 cm^2^. Blood samples (0.5 ml) were collected from retro-orbital plexus into heparinized tubes at 0.5, 1, 1.5, 4, 8, 12, and 24 h after administration. Blood was immediately centrifuged for plasma at 3000 rpm for 10 min. Plasma samples were collected at −70 °C and kept frozen until analysis.

### Chromatographic conditions

Plasma samples were analyzed for FVS adopting a modified liquid chromatography–mass spectrometry (LC–MS/MS) method (Gonzalez et al., 2010). An aliquot of 25 µl of the samples was injected into a Shimadzu Prominence (Shimadzu, Kyoto, Japan) series LC system using Agilent zorbax C18 column (4.6 × 50 mm^2^) with 5 µm particle size. Analysis was carried out at room temperature. The isocratic mobile phase consisted of acetonitrile and 0.01% ammonium acetate (80:20, v/v). This was delivered at a flow rate of 1.1 ml/min into the mass spectrometer’s electrospray ionization chamber. Quantitation was achieved by MS/MS detection in negative ion mode for both FVS and FVS D6 internal standard (IS), using API-4000 mass spectrometer, equipped with a Turbo ionspray^TM^ interface at 500 °C. The compound parameters as declustering potential, collision energy, entrance potential and collision exit potential were –95, –22, –10, and –29 V for FVS and –95, –24, –10, and –23 V for IS, respectively. Detection of the ions was performed in the multiple reactions monitoring (MRM) mode, monitoring the transition of the *m/z* 410.11 precursor ion to the *m/z* 348.2 for FVS and *m/z* 416.18 precursor ion to the *m/z* 354.2 for IS. The analytical data were processed by Analyst software (Version 1.6).

### Samples preparation for analysis

The frozen plasma samples were left to be thawed at room temperature. A liquid–liquid extraction procedure was performed. Plasma samples (500 µl) were mixed with 100 µl of IS (200 ng/ml) then 5 ml of ethyl acetate was added. After vortex mixing for 1 min, the mixture was centrifuged for 10 min at 3000 rpm. The clear supernatant layer was evaporated in vacuum concentrator. The residue was reconstituted in 200 µl mobile phase and injected into the LC apparatus.

### Data analysis

Pharmacokinetic parameters were estimated from plasma data utilizing Excel Add-Ins program, PK Solver software (Zhang et al., 2010). Non-compartmental pharmacokinetic model was adopted for computing the maximum drug concentration (*C*_max_, ng/ml) and time required to attain this concentration (*T*_max_, h) from each rat plasma concentration–time curve. Trapezoidal rule method was applied for calculation of the area under the curve (AUC) from 0 to 24 (AUC_0–24_, ng h/ml) and from 0 to infinity (AUC_0–∞_, ng h/ml).

### *In vivo* study in complete Freund's adjuvant-induced RA in rats

#### Animals

Forty adult female Wistar rats weighing 200–250 g, obtained from animal facility of Nahda University (Beni-Suef, Egypt), were acclimatized to laboratory conditions for two weeks before starting of the study in the air-conditioned pathogen-controlled animal room of Nahda University. The animals were grouped randomly and housed in polyacrylic cages (eight animals per cage) and maintained under standard laboratory conditions (temperature 25 ± 2 °C) with 12/12 h dark and light cycle and allowed free access to standard forage and tap water *ad libitum*. The study was conducted with approval from our institutional animal ethics committee of Beni-Suef University. All efforts were done to reduce the number of animals to the minimum and to decrease the suffering of animals.

### Experimental design

Rats were divided into five groups of eight rats each and designated as follows: group I: a normal control group where rats received only vehicles. Induction of CFA-induced RA in groups II, III, IV, and V; group II: rats injected by CFA for induction of RA using three subcutaneous doses of CFA, each of 0.4 ml, injected in three different limbs with three days interval between every two doses (Ahmed et al., 2017). Group III: rats received standard treatment of 0.1 mg/kg methotrexate (Zhang et al., 2015), administered by oral gavage. Group IV: rats were given FVS 5 mg/kg/day (Haruna et al., 2007) by oral gavage. Group V: rats were treated with FVS-loaded SNVs gel which was applied daily. The application of the gel (1 g) was done on the dorsal surface of each test animal to an area of 4 cm^2^ where hairs were removed by shaving. All treatments were given from 13^th^ day for a period of 27 days. At the end of the study, X-ray of the lower limbs was taken and joint changes were assessed by experienced radiologist. Rats were fasted overnight for 12–14 h and were subjected to mild diethyl ether anesthesia to obtain 8 ml blood from retro-orbital plexus where blood sample was collected then centrifuged at 3000 rpm for 15 min. The clear non-hemolyzed supernatant sera were quickly removed, and kept at –20 °C till used for biochemical assays of serum levels of RF, COMP, ANA, TNF-α, IL-10, MDA, and GSH. Rats were rapidly killed by cervical dislocation soon after blood withdrawal. Whole knee joints with adjacent capsules, tissues, and bones, together with whole spleen were carefully separated. Three of knee joints from different rats in each group were used for western blot analysis and others for histopathological examination.

## Methods

### Assessment of serum biomarkers

Serum RF, COMP, ANA, TNF-α, and IL-10 were assessed using ELISA kits based on manufacturer's protocol. MDA (Ohkawa et al., 1979) and GSH (Beutler et al., 1963) were measured calorimetrically.

### Western blot analysis for detection of p38 MAPK

Synovial tissues were homogenized in radio immunoprecipitation assay (RIPA) lysis buffer PL005 was provided by Bio Basic Inc. (Markham, Canada), supplied with additional protease inhibitor and phosphatase inhibitor buffer cocktail to maintain protein integrity and high biological activity. Homogenization was done for 30 min and then centrifuged at 12,000 rpm for 15 min at 4 °C. The supernatant was collected and used to prepare protein for Western blot analysis. Quantitative protein analysis was performed using Bradford Protein Assay Kit (SK3041) which was provided by Bio Basic Inc. (Markham, Canada). Expression of p38 MAPK was analyzed by Western blot. Thirty μg protein of each sample was heated at 100 °C for 5 min. It was then separated by sodium dodecyl sulfate-polyacrylamide gel electrophoresis (SDS-PAGE) using 10% acrylamide gel and the Bio-Rad minigel system (Hercules, CA). The proteins were electroblotted onto a polyvinylidene difluoride (PVDF) membrane (Bio-Rad, Hercules, CA). Blotting membranes were incubated with 3% bovine serum albumin in tris-buffered saline with Tween (TBST) (10 mmol/l Tris (pH 7.5), 150 mmol/l NaCl, 0.05% Tween-20) and probed with corresponding primary antibodies of anti-p38 MAPK (CST, Beverly, MA) at 4 °C overnight at a dilution 1:1000. PBS used to wash blotting membranes three times and then incubated with horseradish peroxidase-coupled secondary anti IgG monoclonal antibody (Santa Cruz Biotechnology, Santa Cruz, CA) for 2 h at room temperature. Membranes were exposed to films and the bands were visualized using an enhanced chemiluminescence reagent (Amersham^TM^, GE Healthcare, Amersham, UK). These bands were quantitated by densitometry (UVP, Upland, CA). β-actin was used as an internal control for equal protein loading. For each protein, Western blots were repeated three times.

### X-ray radiographic assessment

Radiographs of lower limbs were taken using an X-ray machine with a 0.5 mm focal spot, distance of the focal film was 61 cm, and exposures were 30 s at 45 kVp and 3 mA. Joint changes were assessed based on joint space and deformity. Radiographs were analyzed by an experienced radiologist who was blinded to treatment groups.

### Histopathological study

Whole knee joints with adjacent capsules, tissues and bones together with whole spleen were carefully separated, washed in saline and preserved in 10% formalin solution, sectioned and stained with standard H&E dye as described by Bancroft & Gamble (2008). Sections were investigated by the aid of experienced pathologist.

### Statistical analysis

All numerical data were expressed as mean ± SD. Statistical analysis was performed using one-way ANOVA followed by Tukey’s post hoc test by the aid of the computer software program SPSS 22 (SPSS, Chicago, IL), where the value of *p* < .05 was considered statistically significant.

## Results and discussion

### Factorial design analysis

The applied design was a 2^4^ full factorial design with subsequent statistical analysis utilizing Design-Expert^®^ software. Levels of each factor were based on preliminary trials and possibility of formulating SNVs at the tested values. The picked model was two-factor interaction (2 FI). Adequate precision measured the signal-to-noise ratio to ensure that the model can be applied to navigate the design space (De Lima et al., 2011). A ratio higher than 4 is recommendable which was perceived in all measured responses. Additionally, it is eligible that values of adjusted and predicted *R*^2^ to be in a close accordance in an attempt to be in a plausible harmony (Annadurai et al., 2008). The experimental runs and measured responses are outlined in Table 1. Also, the analysis of variance of a calculated model for a measured response such as effects, degrees of freedom, *F*-ratio, and *p* value for all the factors and interactions is shown in Table S2 (supplemental file).

### FVS entrapment efficiency

For quantification of FVS amount loaded into SNVs, the EE% of the developed formulations was evaluated. The EE% of all SNVs is displayed in Table 1, where EE% oscillated between 34.27 ± 2.15 and 80.07% ± 3.10. Residual analysis and ANOVA assured the suitability of the employed model where the predicated *R*-squared (0.9046) and the adjusted one (0.9721) was close to each other. The adequacy/precision ratio was 23.21, evidencing the potential of the signal to navigate the design space.

Accordingly, the EE% of FVS-loaded SNVs was significantly influenced by the four independent factors (*p* < .05). The regression equation involving the impact of the independent variables on the EE% (Y_1_) in terms of coded values is donated by Equation (3). From the regression coefficients, the Span type (A), EA type (B), and sonication time (D) had a noticeable negative effect on EE% values. On the contrary, EA concentration (C) exerted a significant positive impact on EE%.

(3) EE%=56.69−3.29A−1.98B+6.77C−11.80D−0.13AB −0.078AC−0.38AD−0.15BC−0.33BD−2.00CD

**Figure 1.**
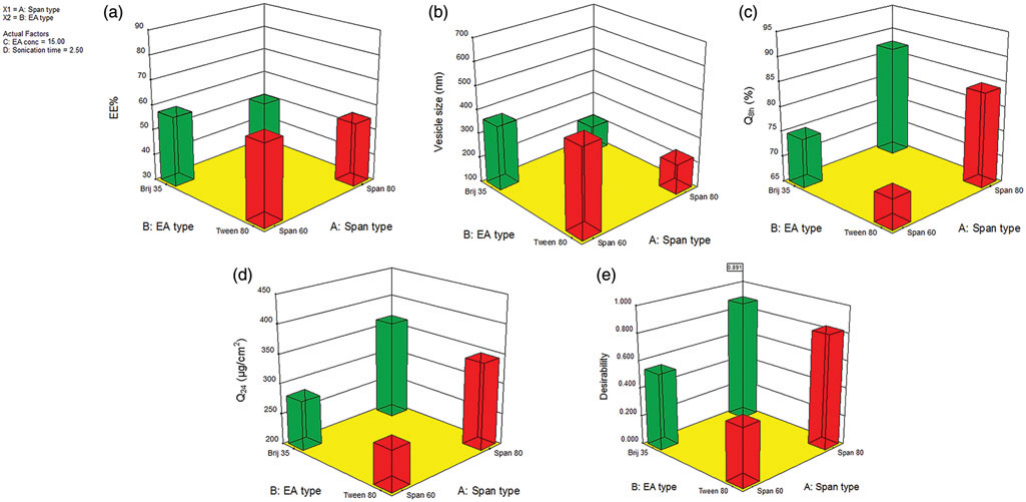
Response surface plot for the effect of Span type (A), EA type (B) at the middle levels of the 3^rd^ and 4^th^ variables (EA concentration and sonication time) on (a) EE%, (b) vesicle size, (c) Q_8h_, (d) Q_24_, and (e) desirability of the developed SNVs dispersions.

The combined effect of the two independent variables (Span type and EA type) on the EE% of FVS-loaded SNVs at the middle levels of the 3^rd^ and 4^th^ variables (EA concentration and sonication time) is demonstrated in Figure 1(a). The EE% values of Span 60-contained SNVs formulations were superior to those of Span 80, despite having equal alkyl chains length, which might be succumbed to the higher phase transition temperature of Span 60 that offers greater drug encapsulation (Yoshioka et al., 1994). Furthermore, the existence of unsaturated alkyl chain in Span 80 structure could give rise to more permeable SNVs membrane with subsequent decrease in EE% compared with Span 60-contained SNVs formulations (Akbari et al., 2015).

Similarly, Tween 80-based SNVs formulations exhibited significant higher EE% than those contained Brij 35, *p* < .05. A plausible explanation may be the difference in the length of the alkyl chain which in turn affects the HLB value of the surfactant and directly affects the EE% (Ruckmani & Sankar, 2010). SNVs fabricated using Tween 80 with the lower HLB values (15) manifested higher EE% compared with SNVs based on Brij 35 possessing the higher HLB values (16.9), incorporating the same FVS amount to load the SNVs. Therefore, the EE% values of SNVs raised as HLB of EA was lowered (ElMeshad & Mohsen, 2016). Comparable results were introduced by Guinedi et al. (2005) who declared that the higher the HLB of the EA, the lower the EE% would be.

There was a positive impact of EA concentration on FVS EE% which could be explained on basis of the aptitude of the EA to endow more space to hold extra drug and a monomolecular EA layer might be created to stabilize the vesicles' interface (Aboud et al., 2018). These findings are in a close agreement with that presented by Al-Mahallawi et al. (2017) who claimed that 20% w/w EA was the optimum concentration for the drug entrapment into SNVs.

It was also clear that sonication time exerted a prominent influence on EE% values of FVS-loaded SNVs. Exposure of SNVs to 5 min sonication significantly reduced FVS EE% (*p* < .05). This could be ascribed to perturbation and re-aggregation of vesicles with concomitant evasion of a significant amount of the drug to the external aqueous milieu comprising the EA and hence, kept in the aqueous milieu *via* micellar solubilization instead of being encapsulated within SNVs. This result is in harmony with that of Andersen et al. (2013) who reported reduced EE% with increased sonication time upon formulation of chitosomes and pectosomes for metronidazole delivery.

### Vesicles size

As depicted in Table 1, the mean vesicle size of the SNVs fluctuated from 167.83 ± 11.73 nm to 692.57 ± 17.03 nm. In general, EAs-contained vesicles are usually spherical with minimal tendency of agglomeration and hence, possessing small particle diameters (Aboud et al., 2019). PDI values of all SNVs formulations ranged between 0.27 and 0.49 which could be conceived as an acceptable mid-range indicating a good size distribution and implying homogeneity of the formulations (Cho et al., 2014).

Factorial statistical analysis divulged the potential of mathematical model to scrutinize the significance influence of the independent variables on the SNVs size (Y_2_). For complying the ANOVA postulation, the Box–Cox plot of vesicle size suggested inverse square root response transformation with lambda = –0.5. The recommended model after transformation for vesicle size was 2 FI with adjusted *R*^2^ of 0.9573 that was in a close proximity to the predicted one of 0.8543. The transformed regression equation relating the response variation to the four factors in coded values was:

(4) 1/Sqrt(Vesicle size)=0.058+9.133E−003A+1.984E−003B +5.439E−003C+2.730E−003D−9.133E−004AB −6.183E−004AC+5.323E−005AD−6.130E−004BC +4.057E−004BD+6.109E−004CD

Our results revealed a significant influence of the examined terms on SNVs mean size (*p* < .05). As illustrated in Figure 1(b), the average size of Span 60-based SNVs was higher as compared with that of Span 80-based vesicles. Presumably, the lower the HLB of the surfactant, the smaller the vesicle size produced. Thus, Span 80-based SNVs (HLB 4.3) were smaller sized than those contained Span 60 (HLB 4.7). The observed correlation between the surfactant HLB and vesicle size could be imputed to decrement in surface energy ensued from hydrophobicity increment, hence leading to formation of smaller vesicles (Khallaf et al., 2019). Yoshioka et al. (1994) reported similar findings on estimating the characteristics of different Spans contained niosomes.

Similar to the Span type effect, the EA type had a significant effect on the size of FVS-loaded SNVs (*p* ˂ .05). As demonstrated in Figure 1(b), smaller sized SNVs were evinced in case of Brij 35 rather than Tween 80 which could be referred to the variation in the alkyl chain length of these EAs. Indeed, smaller vesicles are formed upon inclusion of EAs possessing shorter alkyl chains and vice versa (Uchegbu & Florence, 1995). In our study, Brij 35 (C12) has shorter alkyl chain and therefore, less bulky structure relative to Tween 80 (C18), thus, smaller vesicle diameters were yielded. These data are parallel to that achieved by a previous study (Al-Mahallawi et al., 2017).

It is worth noting that EA concentration had a significant antagonistic influence on the mean SNVs' size (*p* ˂ .05). This can be interpreted in the light of lowering the interfacial tension at higher surfactant concentration with subsequent development of smaller vesicles. On the contrary, lower concentration of EA may be disabled to overlay the whole vesicle circumference. Thus, agglomeration of some vesicles might occur with reduction in the surface area to the point that the EA concentration could endure the entire aggregate's surface and thereby, formation of relatively stable larger particles occurred (Al-Mahallawi et al., 2017). Mahmoud et al. (2017) shared similar results in their investigation on the formulation of atorvastatin-loaded nanovesicular systems.

Expectedly, a significant reduction in the vesicle size was attained upon sonication of FVS-loaded SNVs for 5 min (*p* ˂ .05). This may be resulted from exposition of the vesicles to ultrasonic waves leading to dissipation of SNVs into smaller sizes (Elsherif et al., 2017). Such results endorsed with previous literature reports (Shukla et al., 2014; El-Say et al., 2016).

#### *In vitro* drug release studies

The release profiles of FVS from the fabricated SNVs dispersions and its aqueous solution in Sorensen's phosphate buffer pH 5.5 are graphically illustrated in Figure S1a,b (supplemental file). The % FVS released from the aqueous solution within 3 h was approximately 92.14%; pointing out that the inspected dialysis membrane did not halt the drug release. The % FVS release from SNVs after 8 h ranged from 67.23 ± 11.75 to 92.08 ± 3.00% as denoted in Table 1. The release profiles of FVS among various SNVs were obviously biphasic modes where rapid release of the superficially adsorbed drug was perceived within the first 2 h (initial phase) accompanied by a prolonged release manner. The explored model was statistically significant concerning residual analysis and ANOVA with adequacy/precision ratio of 30.13 elucidating adequate signal. The quantitative effect of independent variables on the release of FVS-loaded SNVs in coded values is represented by Equation (5).

(5) Q8h=79.43+6.47A+1.86B+3.54C+0.76D−0.12AB +0.29AC−0.024AD+0.31BC−0.038BD+0.018CD

The investigated independent variables revealed a significant impact on the Q_8h_ among the various dispersions (*p* < .05) (Figure 1(c)). As regards to Span type, it was observed that the Q_8h_ values of Span 80-composed SNVs were significantly higher than those of Span 60-composed SNVs (*p* < .05). As has already been outlined, rapid release of Span 80-based SNVs could be due to its unsaturated alkyl chain which in turn brought FVS leakage out. Additionally, lower phase transition temperature of Span 80 may be another reason for the manifested boosted release. The phase transition temperature of Span 60 is 53 °C versus −12 °C for Span 80 (Mokhtar et al., 2008). Since the release study was performed at 32 ± 0.5 °C, lower release rates of Span 60-based vesicles might be due to its higher transition temperature virtually rendering them in a robustly ordered gel condition. These observations coincide with that suggested by Elsherif et al. (2017) who fabricated terbinafine hydrochloride-loaded SNVs.

A notable finding is that, Brij 35-decorated vesicles yielded a significantly higher release rates compared to Tween 80-decorated vesicles (*p* < .05). This could be attributed to the alkyl chain length-dependent release pattern of SNVs where the lower the chain length, the higher the release rate (Devaraj et al., 2002). Furthermore, the smaller particle size of Brij 35-based SNVs could offer greater surface area exposed to the release milieu and hence, augmented the drug release.

According to statistical analysis of the release data, both EA concentration as well as sonication time had a significant positive impact on Q_8h_ of FVS-loaded SNVs (*p* < .05). This positive correlation between sonication time as well as EA concentration and the Q_8h_ might be claimed to the vesicle size. As the percentage of the drug dissolved in the aqueous milieu at any time is oppositely proportional to the vesicles' diameter. Thus, the smaller vesicles produced at high concentration of EAs could lessen the diffusional distance of the drug with subsequent promotion of drug release rates (Wacker, 2013).

Mathematical analysis of FVS release data disclosed that the drug release from most of the formulated dispersions obeyed Higuchi kinetics release model, demonstrating a diffusion controlled mechanism. Several studies declared that drug-based vesicular systems impart a controlled release fashion complying with Higuchi's square root model (Mahmoud et al., 2017; Aboud et al., 2018).

For further interpretation of FVS release kinetics, the Korsmeyer–Peppas model was employed which can shed light on other mechanisms of drug release. In the Korsmeyer–Peppas equation, the *n* values for Fickian (diffusional) and zero-order release kinetics equal 0.5 and 1, respectively, while 0.5 < *n* < 1 for non-Fickian (anomalous) release. In our study, the *n* values for the different dispersions ranged from 0.53 to 0.81, manifesting non-Fickian drug diffusion and anomalous drug release pattern in which amalgamation of drug diffusion with the lipid bilayers' distention might occur (El-Nabarawi et al., 2016).

### *Ex vivo* permeability study

Regarding the implementation of SNVs as a transdermal platform, *ex vivo* diffusion studies were scrutinized to prognosticate their *in vivo* performance. *Ex vivo* skin permeation profiles of FVS-loaded SNVs *via* rat skin relative to FVS solution were represented in Figure S2a,b (supplemental file). The developed SNVs demonstrated significant higher skin permeation compared with drug solution comprising equal quantity of FVS (*p* < .05). From Figure S2 (supplemental file), it is noticed that only 211.9 ± 17.01 µg/cm^2^ of FVS solution was permeated *via* rat skin over 24 h; meanwhile, the cumulative amount of FVS permeated from SNVs ranged from 229.44 ± 20.45 to 460.59 ± 20.01 µg/cm^2^. Collectively, the calculated permeation parameters of the inspected FVS-loaded SNVs *via* rat skin were summarized in Table S3 (supplemental file). The investigated SNVs exhibited transdermal flux values in the range of 9.56 ± 0.85 to 19.18 ± 0.83 µg/cm^2^ h versus 8.81 ± 0.48 µg/cm^2^ h for the control FVS solution. Therefore, the obtained results emphasized the preponderance of SNVs in both sustaining FVS release and promoting its skin diffusion from 1.79- to 4.46-fold greater than FVS solution. Statistical analysis with ANOVA disclosed that the sequential model recommended for estimating the Q_24_ response was 2 FI with adjusted *R*^2^ value of 0.994 designating that nearly 99% of the entire variations in the transdermal Q_24_ could be elucidated by the model. Thus, the Q_24_ could be allied to the four factors by the following equation in terms of coded value:

(6) Q24=316.69+41.20A+9.08B+25.38C+11.44D+1.08AB +7.02AC+1.33AD+1.39BC−1.85BD+0.97CD

The outcome of the formulation variables on the Q_24_ of FVS-loaded SNVs is illustrated in Figure 1(d). The four independent factors significantly affected the Q_24_ parameter (*p* < .05). It is observed that all terms had a positive regression coefficient, pointing out the positive impact on the transdermal diffusion upon raising their levels. This boosted transdermal FVS delivery from the fabricated SNVs might be due to the potential of the vesicular carrier to present FVS as a minute colloidal dispersion with larger surface area and hence, diminishing FVS diffusional path length across the skin (Fahmy, 2015). Additionally, tailoring of SNVs with EAs might be another reason for their ameliorated skin diffusion where EAs could increase SNVs deformability and accordingly, enhance the vesicular capacity for retaining and binding water upon application under non-occlusive state to shun dehydration (El Zaafarany et al., 2010). Thus, SNVs can afford deep migration to water opulent strata holding drug molecules to guarantee appropriate hydration condition. Moreover, EAs possess high propensity for extremely curved framework which permits SNVs to succumb stress-dependent modulation of their current assembly to conquer their movement reluctance across the confining skin channel and warrant noninvasive and reproducible drug transport (Cevc & Blume, 2003).

In our study, Brij 35/Span 80-contained SNVs were more readily permeated than other SNVs contained Tween 80/Span 60 mixture. The observed variation in skin permeation parameters might be claimed to difference in the particle size of the developed SNVs. Interestingly, the highest Kp and Q_24_ were achieved in formulation S16 containing Brij 35/Span 80 in the concentration of 20% w/w (relative to EA/Span 80 total weight, with sonication for 5 min) which could be referred to the conferred flexibility of the vesicles with subsequent higher permeation accomplished at the adequate concentration of this combination. On the contrary, the lowest permeation parameters were procured in formulation S1 containing Tween 80/Span 60 in the concentration of 10% w/w (with respect to EA/Span 60 total weight, without sonication) which possessed the largest vesicle diameter. These results run in parallel with those obtained from the *in vitro* release studies.

### Selection of the optimal formulation

The aim of pharmaceutical processing optimization is the tailoring of the formulation independent variables to yield a product of high quality with optimal physicochemical characteristics (Al-Mahallawi et al., 2017). Therefore, application of the desirability function was employed for selection of the optimized dispersion from the fabricated 16 SNVs dispersions according to the proposed 2^4^ full factorial design. The desirability constraints for the optimized dispersion (maximizing EE%, Q_8h_ and Q_24_ as well as minimizing vesicle size) were scrutinized with overall desirability value of 0.891, as illustrated in Figure 1(e). The optimal formulation was fabricated using Brij 35/Span 80 in the concentration of 20% w/w (with respect to EA/Span 80 total weight, without sonication) and exhibited EE% of 71.28 ± 2.05%, vesicle size of 201.54 ± 9.16 nm, Q_8h_ of 89.45 ± 3.64%, and Q_24_ of 402.55 ± 27.48 (µg/cm^2^). As depicted in Table S4 (supplemental file), the observed values of the optimal formulation were highly comparable to the predicted ones manifesting a small percentage of prediction error that fluctuated from 1.49 to 4.56% for different responses, emphasizing the adequacy and fitness of the proposed mathematical model for speculation of dependent responses. Thereby, this formulation was elected for further investigation.

### Morphology of FVS vesicles

**Figure 2.**
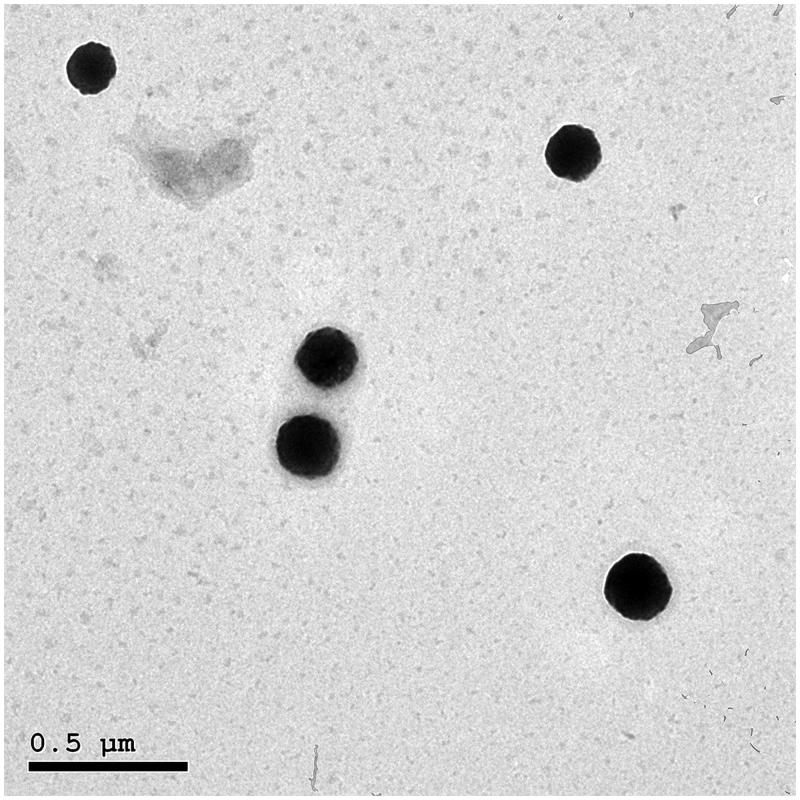
Transmission electron micrograph of the optimal SNVs formulation.

The morphology of the optimal SNVs dispersion was explored *via* TEM imaging (Figure 2). The speculated vesicles were spherically and uniformly shaped, well dispersed with no aggregation noted and having relatively smooth surfaces. Morphological investigation assured the former vesicle size values determined with DLS. TEM results emphasized the fitting of the optimal formulation to breach the SC barrier.

### Differential scanning calorimetry

DSC thermograms of pure FVS, Span 80, Brij 35 as well as the optimized SNVs formulation are illustrated in Figure S3 (supplemental file). The DSC thermogram of Brij 35 revealed an endothermic peak at 44.45°C representing its gel–liquid transition temperature. The DSC thermogram of FVS exhibited an endothermic peak at 64.07 °C which is the same as reported previously in the literature for FVS (Li et al., 2018). The DSC thermogram of the optimized SNVs formulation disclosed FVS melting endotherm subsidence. The extinction of the distinguishing endothermic peak of FVS as well as broadening and/or shifting of the endotherms of surfactant bilayers propose potential interaction of drug with bilayer components that may be associated with numerous lattice defects with amorphous regions formation in which the drug is resided and can account for the enhanced entrapment of FVS into these SNVs in an amorphous state (ElMeshad & Mohsen, 2016).

### SNVs stability

The physical stability of the optimized SNVs formulation was assessed following storage at 4 °C over a period of 3 months. The optimal SNVs formulation manifested good physical stability with no visible change in the appearance. As depicted in Table S5 (supplemental file), the FVS EE%, particle size, and size distribution exhibited insignificant change after 3 months of storage (*p* > .05), confirming kinetic stability of the stored SNVs during this period. The enhanced physical stability observed ensures the adequacy of Span 80 and Brij 35 amalgamation in the lipid vesicular carrier.

### Skin irritation study

Histopathological examination denoted insignificant alteration in skin structure of SNVs gel-treated group compared to the untreated skin in the normal control group indicating the tolerability of the applied FVS-loaded SNVs transdermal gel, Figure S4 (supplemental file). Additionally, none of the adverse reactions signs as skin erythema or irritation was observed in any of the rats at the gel application site over the treatment course.

### Pharmacokinetic studies

The pharmacokinetic study results demonstrated well release and permeation of FVS from the transdermal SNVs gel as compared with either the oral solution or the traditional gel (Table 2). The maximum concentration of 416.61 ± 25.92, 339.88 ± 27.76, and 120.50 ± 17.52 ng/ml was accomplished at 1.17 ± 0.26, 7.33 ± 1.03, and 3.58 ± 1.02 following administration of oral solution, SNVs gel and traditional gel, respectively. The AUC_0–∞_ values were 1131.42 ± 40.96, 3156.93 ± 180.06, and 687.74 ± 66.01 ng h/ml for the oral solution, SNVs gel and traditional gel, respectively. Comparison of *C*_max_, *T*_max_, and AUC profiles was pursued. *C*_max_ was significantly higher for oral route than transdermal route (*p* ˂ .05). Conversely, *T*_max_ values were significantly higher for transdermal administrations than oral administration (*p* < .05). This delayed behavior of transdermally applied gels could be attributed to the SC fence that might retard FVS permeation from both of SNVs and traditional gels while the oral solution is an immediate release preparation. A notable finding is that SNVs gel disclosed a significantly retarded *T*_max_ (7.33 h) than the traditional gel (3.83 h) which might be claimed to the propensity of SNVs gel, contrasting the traditional gel, to execute as a repository; thereby, proposing a platform for sustained release delivery (Mahmoud et al., 2017). Furthermore, the mean value of AUC_0−∞_ for the SNVs gel was 2.79- and 4.59-fold significantly higher than the parallel mean value for the oral solution as well as the traditional gel, respectively (*p* < .05). The SNVs gel was superior to both the oral solution and the traditional gel in presenting FVS to the systemic circulation. This could be attributed to eschewing the first-pass effect attendant with peroral FVS administration and the penetration ameliorating properties of the fabricated ultradeformable nanocarrier through attenuation of the barrier function of the SC as well as perturbation of the lipid bilayer and expedition of leakage of proteins and lipids from the skin layers. In the current investigation, the relative bioavailability of FVS administered by the transdermal SNVs gel was found to be 279.02% compared with the oral solution.

**Table 2.** Mean pharmacokinetic parameters for FVS in rat plasma following administration of oral solution, SNVs gel and traditional gel.

Pharmacokinetic parameter	Mean ± SD		
	Oral solution	SNVs gel	Traditional gel
*C*_max_ (ng/ml)	416.61 ± 25.92	339.88 ± 27.76^a^	120.50 ± 17.52^a^^,^^b^
*T*_max_ (h)	1.17 ± 0.26	7.33 ± 1.03^a^	3.58 ± 1.02^a^^,^^b^
*K*_elim_ (h^–1^)	0.2219 ± 0.0437	0.10678 ± 0.0101^a^	0.1904 ± 0.0145^a^^,^^b^
*t*_1/2_ (h)	3.12 ± 0.42	6.49 ± 0.67^a^	3.64 ± 0.21^a^^,^^b^
AUC_0–24_ (ng h/ml)	1126.91 ± 74.60	3093.82 ± 217.50^a^	683.69 ± 78.38^a^^,^^b^
AUC_0–∞_ (ng h/ml)	1131.42 ± 40.96	3156.93 ± 180.06^a^	687.74 ± 66.01^a^^,^^b^
*F*_rel_ (%)	–	279.02	60.79

Values are means ± SD, with the number of animals = 6 for each group. Using one-way ANOVA followed by Tukey’s post hoc test.

a *p* < .05 versus oral FVS solution.

b *p* < .05 versus SNVs gel.

### *In vivo* study in CFA-induced RA in rats

In the current study, a new preparation of FVS in the form of SNVs transdermal gel was evaluated for its possible beneficial effects against CFA-induced RA in adult female albino rats, in comparison with the conventional FVS and the standard agent, methotrexate. Transdermal formulation could ensure that drug levels neither fall below the minimum effective concentration nor exceed the maximum effective concentration as it maintains drug concentration within the therapeutic window for prolonged period of time (Alkilani et al., 2015). In the present study, FVS-loaded SNVs transdermal gel significantly decreased (*p* < .05) specific rheumatoid markers; RF and COMP by 64.05% and 73.53%, respectively, as compared to arthritis control group as shown in Table 3. Ahmed et al. (2015) revealed that simvastatin significantly decreased RF and COMP in RA animal model. COMP regulates the cellular proliferation, apoptosis, and cellular attachment in the cartilaginous tissue, being one of the essential components of the extracellular matrix of the cartilage (Das et al., 2015).

**Table 3.** Biochemical analysis in different studied groups.

Parameter	Normal control	Arthritis control	Arthritis + MTX	Arthritis + FVS	Arthritis + FVS- loaded
% of change	(group 1)	(group 2)	(group 3)	(group 4)	SNVs gel (group 5)
RF (IU/ml)	1.74 ± 0.47	12.60 ± 1.56^a^	4.34 ± 0.34^a^^,^^b^	6.33 ± 0.21^a^^,^^b^	4.53 ± 0.29^a^^,^^b^
% of change	–	(624.14%)	(–65.56%)	(–49.76%)	(–64.05%)
COMP (ng/ml)	1.47 ± 0.29	15.87 ± 1.42^a^	3.40 ± 0.35^a^^,^^b^	5.23 ± 0.32^a^^,^^b^	4.20 ± 0.40^a^^,^^b^
% of change	–	(979.59%)	(–78.58%)	(–67.04%)	(–73.53%)
ANA (ng/ml)	1.82 ± 0.22	19.53 ± 2.90^a^	5.16 ± 0.82^b^	8.20 ± 0.70^a^^,^^b^	6.06 ± 1.00^a^^,^^b^
% of change	–	(973.08%)	(–73.58%)	(–58.01%)	(–68.97%)
TNF-α (pg/ml)	34.53 ± 1.76	102.13 ± 14.77^a^	44.87 ± 3.01^b^	65.83 ± 6.93^a^^,^^b^	46.33 ± 2.21^b^
% of change	–	(195.77%)	(–56.07%)	(–35.54%)	(–54.64%)
IL-10 (pg/ml)	124.33 ± 2.21	48.73 ± 12.56^a^	108.73 ± 6.33^b^	99.83 ± 6.93^a^^,^^b^	109.50 ± 3.82^b^
% of change	–	(–60.81%)	(123.13%)	(104.86%)	(124.71%)
MDA (nmol/mg)	9.33 ± 0.75	46.07 ± 2.86^a^	13.43 ± 1.35^b^	12.23 ± 1.55^b^	10.07 ± 0.75^b^
% of change	–	(393.78%)	(–70.85%)	(–73.45%)	(–78.14%)
GSH (mmol/mg)	61.43 ± 2.72	28.77 ± 2.29^a^	50.77 ± 4.35^a^^,^^b^	47.23 ± 2.91^a^^,^^b^	53.87 ± 4.19^b^
% of change	–	(–53.17%)	(76.47%)	(64.16%)	(87.24%)

MTX: methotrexate; FVS: fluvastatin; SNVs: spanlastic nanovesicles; RF: rheumatoid factor; COMP: cartilage oligomeric matrix protein; ANA: antinuclear antibody; TNF-a: tumor necrosis factor-alpha; IL-10: interleukin-10; MDA: malondialdehyde; GSH: glutathione reduced.

Each value represents the mean of 8 values ± SD. Statistical analysis was performed using one-way ANOVA test followed by Tukey’s post hoc test. Differences between group means were considered significant at *p* < .05. Percentage (%) changes were calculated by comparing arthritic control group with normal control and arthritic-treated groups with the arthritic control group.

a Significantly different from normal control group value at *p* < .05.

b Significantly different from arthritic control group value at *p* < .05.

Vervoordeldonk and Tak (2002) claimed that the inflammatory response that occur in RA is mainly mediated by TNF-α and the remarkable decline in IL-10 may indicate severe inflammatory response in arthritic rats since IL-10 attenuates the release of proinflammatory cytokines from activated macrophages and neutrophils, serving as an anti-inflammatory signal (Liu et al., 2011). Our data revealed a robust inflammatory response in arthritis control rats indicated by significant elevation of TNF-α levels, ANA (*p* < .05) by 195.77% and 973.08%, respectively, in addition to significant decrease of IL-10 (*p* < .05) by 60.81% as compared to normal control group as in Table 3. These findings are in line with a previous study (Ahmed et al., 2017). Cytokines are reported to produce antibodies directed against self-antigens found in the joint, including RF, IgG, and ANA as they activate B lymphocytes (Biesen et al., 2014). According to current results, the anti-arthritic potential of FVS transdermal SNVs gel may be attributed to its immunomodulatory and anti-inflammatory effect evidenced by significantly suppressed ANA production by 68.97%, decreased TNF-α production by 54.64% and augmented IL-10 anti-inflammatory signal by 124.71% as compared to arthritis control group (*p* < .05). Additionally, no significance of TNF-α and IL-10 levels (*p* > .05) was observed in FVS transdermal SNVs gel-treated arthritic animals as compared to normal control group and interestingly, these findings were in line with results of methotrexate treated rats emphasizing the anti-inflammatory effect as demonstrated in Table 3. In agreement, Mathew et al. (2013) revealed that FVS alleviates inflammation and oxidative stress in CFA-induced arthritic rats by the downregulation of TNF-α and IL-6. Also Lee et al. (2009) concluded that pretreatment with FVS can suppress the release of serum TNF-α and can also increase serum IL-10 level to protect hemorrhagic shock-induced multi-organ damage in rats.

Moreover, the present findings revealed that RA triggered the activation of MAPKs that play an important role in RA as it is involved in continuous inflammation, synovial proliferation, pannus formation, and joint destruction (Zwerina et al., 2006). More importantly, blockage of p38 MAPK with p38 inhibitor could suppress the chondrocytes apoptosis, decrease the downstream inflammatory cytokine production and prevent the involvement of other inflammatory cells that may result in degradation of bone and cartilage (Sun et al., 2017). Thus, p38 MAPK has been considered by authors of the present study to be an attractive target for drug attenuated inflammatory processes in RA and interestingly, the current study clearly demonstrated that FVS-loaded SNVs gel was able to downregulate the activation of p38 MAPK cascade as demonstrated in Figure S5a (supplemental file) and indicated by not only significant suppression of p38 MAPK by 73.80% (*p* < .05), Figure S5b,c (supplemental file), as compared to arthritis control group but also it decreased p38 MAPK expression significantly (*p* < .05) as compared to arthritic rats treated by conventional FVS, Figure S5b (supplemental file) and more interestingly, it was the only treatment among other used drugs even the standard one (methotrexate), that nearly normalized the level of p38 MAPK as there was no significance (*p* > .05) when compared to normal control group suggesting an important mechanism for mitigation of RA by FVS-loaded SNVs gel. Senokuchi et al. (2005) revealed that statin targets the MAPK families of signal transduction molecules.

Also it is worth mentioning that high lipid peroxidation was an indicator of reduced antioxidant capacity and increased oxidative stress in RA (Suresh et al., 2012). GSH is endogenously synthesized in the liver and considered the first line of defense against peroxidation (Suresh et al., 2012). Accordingly, amelioration of oxidative stress has an important anti-arthritic role. The antioxidant effect of FVS transdermal SNVs gel was evident in the current study as it nearly normalized levels of MDA and GSH; indicated by no significant difference (*p* > .05) as compared to normal control animal group, in addition to significant alleviation of MDA levels by 78.14% and significant increase of GSH by 87.24% (*p* < .05) as compared to arthritis control group as in Table 3. While arthritic animals treated by conventional FVS or methotrexate decreased MDA and increased GSH levels significantly as compared to arthritis control but only MDA level was insignificant (*p* > .05) when compared to normal control group as shown in Table 3. These results suggested that the antioxidant effect of FVS-loaded SNVs is more evident than conventional FVS. Similar results of previous studies reported that FVS mitigated inflammation and oxidative stress in arthritis (Haruna et al., 2007; Mathew et al., 2013). FVS has a strong antioxidant effect as it has been reported to suppress the generation of superoxide anions (Suzumura et al., 1999). Hanayama et al. (2009) demonstrated that FVS but not pravastatin, through a blockade of the classical mevalonate pathway and an antioxidant action, significantly attenuated osteoclast differentiation and activation leading to prevention of osteoporosis.

**Figure 3.**
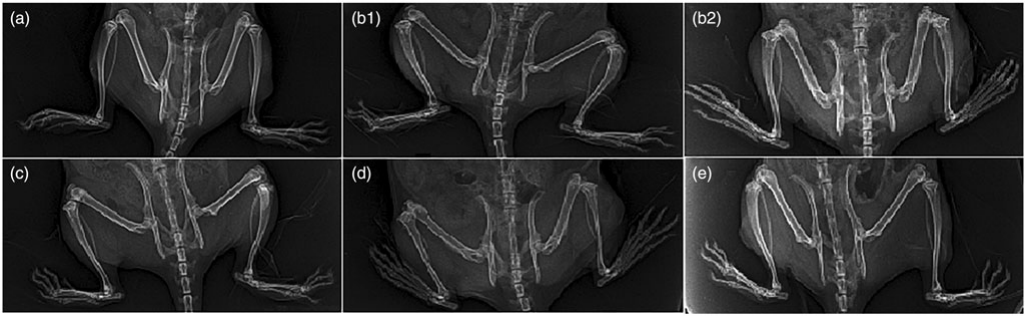
Radiological changes of joints. (a) Normal control group. (b1 and b2) Arthritis control group showing foot deformity, marked knee joint space narrowing and osteopenia. (c) Methotrexate-treated group showing normal joint space, but mild foot deformity. (d) Oral FVS-treated group showing mild narrowing of joint space, mild foot deformity and osteophytes. (e) FVS-loaded SNVs gel-treated group showing relative narrowing of knee joint space with mild foot deformity without osteopenia or osteophytes.

In addition to the above results, Figure 3 depicts the radiological changes in normal, arthritis, and arthritic treated-rats. Arthritis control group showed obvious foot deformity, marked narrowing of knee joint space and osteopenia when compared to normal control group as in Figure 3(b); these results are in accordance with Ruckmani et al. (2018) who reported similar findings. Treatment of arthritic rats with FVS-loaded SNVs gel mitigated these radiological findings of arthritis control group, demonstrated by decreased foot deformity, ameliorated narrowing of joint space (relative narrowing) and there was no osteopenia or osteophytes as shown in Figure 3(e).

**Figure 4.**
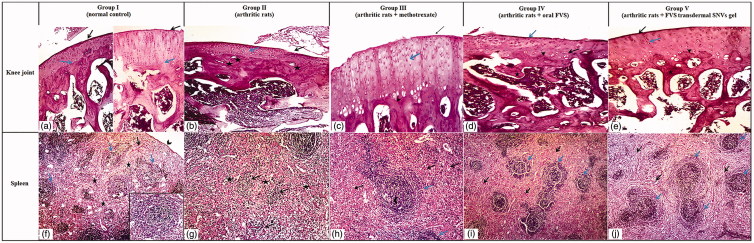
Photomicrographs of articular cartilage and splenic parenchymae of different sections in different groups (H&E ×100). (a–e) Articular cartilage of different groups. (a) Normal control rat section showing a smooth articular surface (black arrow) and a regular tide mark (blue arrow) separating the articular cartilage from the underlying subchondral bone. (b) Arthritis control rat section showing disrupted articular surface (black arrow), cartilage layer was thinner with cell loss, cell cloning and multicellular chondrocyte clusters and overall the cells appeared in a less ordered structure (blue arrow). The subchondral bone invaded the calcified cartilage (stars), top right. (c) Methotrexate-treated arthritic rat section showing slight smooth articular surface (black arrow), thickened articular cartilage with proliferating chondrocytes (blue arrow), hypercellularity and cloning. Regular tide mark can be seen (arrowhead). (d) Oral FVS-treated arthritic rat section showing slightly smooth articular surface (blue arrow) with thinner articular cartilage layer. The subchondral bone (star) can be observed. The proliferating chondrocytes showing hypercellularity, cloning, and slightly ordered distribution (black arrow). Regular tide mark can be seen (arrowhead). (e) FVS-loaded SNVs gel-treated arthritic rat section showing smooth articular surface (black arrow). Thickened articular cartilage and subchondral bone (star) can be observed. The proliferating chondrocytes showing hypercellularity, cloning, and slightly ordered distribution (blue arrow). Regular tide mark can be seen (arrowhead). (f–j) Splenic parenchymae of different sections. (f) Normal control rat section showing thin capsule (arrowhead), trabeculae (black arrow) and white pulp formed of lymphatic follicles (blue arrow) surrounded with red pulp (stars). Inserted box showing lymphatic follicles with central arteriole and well-defined marginal zone. (g) Arthritis control rat section showing marked atrophy of white pulp (black arrow) and red pulp (stars). (h) Methotrexate-treated arthritic rat section showing proliferating lymphatic follicles (white pulp) (blue arrow) surrounded with hyperplastic red pulp (black arrow). Lymphatic follicles showing central arteriole (arrowhead) and well-defined marginal zone. (i) Oral FVS-treated arthritic rat section showing slightly proliferating lymphatic follicles (white pulp) (blue arrow) surrounded with hyperplastic red pulp (black arrow). Lymphatic follicles showing central arteriole and well-defined marginal zone. (j) FVS-loaded SNVs gel-treated arthritic rat section showing slightly proliferating lymphatic follicles (white pulp) (blue arrow) surrounded with hyperplastic red pulp (black arrow). Lymphatic follicles showing central arteriole and well-defined marginal zone.

The previous biochemical and radiological results were confirmed by histopathological findings of the knee joint and spleen where FVS transdermal SNVs gel-treated arthritic rat articular sections showed prominent histopathological improvements of the joint as compared with arthritis control rats demonstrated by smooth articular surface, thickened articular cartilage, and subchondral bone; the proliferating chondrocytes showing hypercellularity, cloning and slightly ordered distribution and also regular tide mark was seen as in Figure 4(a–e). In addition to attenuation of the atrophic changes of white and red pulps of the splenic parenchymae that depicted in arthritis control group indicated by slightly proliferating lymphatic follicles (white pulp) surrounded with hyperplastic red pulp and lymphatic follicles showed central arteriole and well-defined marginal zone, as demonstrated in Figure 4(f–j).

Finally, endothelial dysfunction plays a major role in morbidity of RA during early stages of the disease; however, giving statins at the proper time can have a beneficial effect on the vasculature (Cavagna et al., 2012). Haruna et al. (2007) indicated that FVS had potent vascular protective effects in adjuvant-induced arthritis. FVS was reported to diminish adhesive interaction between monocytes and vascular wall (Niwa et al., 1996). So, according to the current study, transdermal delivery of FVS-loaded SNVs gel will be a promising new formulation having overall favorable effects on the disease course as well as providing efficient and easy route of administration.

## Conclusions

In the present study, SNVs were fabricated as ultradeformable nanocarrier for FVS transdermal delivery for management of RA. The vesicular dispersions were successfully elaborated adopting thin film hydration technique. Optimization of the SNVs formulations was pursued applying a 2^4^ full factorial design. The optimum SNVs formulation containing Brij 35/Span 80 in the concentration of 20% w/w (with respect to EA/Span 80 total weight) exhibited adequate EE%, small vesicle size, spherical morphology, a sustained release profile over 8 h and ameliorated permeation characteristics across rat skin. The pharmacokinetic study divulged substantially enhanced bioavailability of the optimum FVS-loaded SNVs gel of about 2.79-fold and prolonged *t*_1/2_ to about 6.49 ± 0.67 h compared with oral drug solution. Additionally, the potent ameliorating effects of the developed transdermal formulation on CFA-induced arthritis in experimental rats were evidenced by significant decrease in rheumatoid markers, in addition to anti-inflammatory, antioxidant effects and most importantly, significant suppression of p38 MAPK expression as compared to arthritic control and arthritic treated group with oral FVS. Thus, the obtained results unravel the potential of SNVs for promoting the non-invasive transdermal delivery of FVS and donating platform for RA treatment.

## Supplementary Material

Supplemental Material
