# Supplementary material for: Transdermal delivery of fluvastatin sodium via tailored spanlastic nanovesicles: mitigated Freund's adjuvant-induced rheumatoid arthritis in rats through suppressing p38 MAPK signaling pathway
Source: Drug Deliv. 2019 Nov 18;26(1):1140–54. doi: 10.1080/10717544.2019.1686087 (PMC6882467; doi:10.1080/10717544.2019.1686087)
Supplement: Supplemental Material [file IDRD_A_1686087_SM7960.docx]

Table (S1): Variables and their corresponding levels in the employed 2^4^ full factorial design for FVS-loaded SNVs

| Variable | Design level | |
| --- | --- | --- |
|  | Low (-1) | High (+1) |
| Independent variables |  |  |
| A = Span type | Span 60 | Span 80 |
| B = EA type | Tween 80 | Brij 35 |
| C = EA concentration (% w/w)^a^ | 10 | 20 |
| D = Sonication time | 0 | 5 |
| Dependent variables | Constraints |  |
| Y_1_ = EE% | Maximize |  |
| Y_2_ = Vesicle size (nm) | Minimize |  |
| Y_3_ = Q_8h_ (%) | Maximize |  |
| Y_4_ = Q_24_ (µg/cm^2^) | Maximize |  |

EA: edge activator; EE%: entrapment efficiency percent; Q_8h_: cumulative release after 8 h; Q_24_: cumulative amount permeated/unit area in 24 h.

a% (w/w) of the EA with respect to Span/EA total weight.

Table (S2): Analysis of variance of the final models for measured responses

| **Parameters** | **SS** | **DF** | **MS** | **F** | **p-value** | **Parameters** | **SS** | **DF** | **MS** | **F** | **p-value** |
| --- | --- | --- | --- | --- | --- | --- | --- | --- | --- | --- | --- |
| **EE%** |  |  |  |  |  | **Vesicle size** |  |  |  |  |  |
| Model | 3266.17 | 10 | 326.62 | 53.19 | 0.0002 | Model | 2.024E-003 | 10 | 2.024E-004 | 34.65 | 0.0005 |
| A | 173.47 | 1 | 173.47 | 28.25 | 0.0032 | A | 1.335E-003 | 1 | 1.335E-003 | 228.44 | < 0.0001 |
| B | 62.87 | 1 | 62.87 | 10.24 | 0.0240 | B | 6.301E-005 | 1 | 6.301E-005 | 10.79 | 0.0219 |
| C | 733.73 | 1 | 733.73 | 119.49 | 0.0001 | C | 4.734E-004 | 1 | 4.734E-004 | 81.03 | 0.0003 |
| D | 2227.45 | 1 | 2227.45 | 362.74 | < 0.0001 | D | 1.193E-004 | 1 | 1.193E-004 | 20.42 | 0.0063 |
| AB | 0.28 | 1 | 0.28 | 0.046 | 0.8393 | AB | 1.335E-005 | 1 | 1.335E-005 | 2.28 | 0.1911 |
| AC | 0.098 | 1 | 0.098 | 0.016 | 0.9046 | AC | 6.116E-006 | 1 | 6.116E-006 | 1.05 | 0.3531 |
| AD | 2.34 | 1 | 2.34 | 0.38 | 0.5642 | AD | 4.533E-008 | 1 | 4.533E-008 | 7.760E-03 | 0.9332 |
| BC | 0.35 | 1 | 0.35 | 0.056 | 0.8220 | BC | 6.012E-006 | 1 | 6.012E-006 | 1.03 | 0.3569 |
| BD | 1.79 | 1 | 1.79 | 0.29 | 0.6125 | BD | 2.634E-006 | 1 | 2.634E-006 | 0.45 | 0.5317 |
| CD | 63.80 | 1 | 63.80 | 10.39 | 0.0234 | CD | 5.972E-006 | 1 | 5.972E-006 | 1.02 | 0.3584 |
| Residual | 30.70 | 5 | 6.14 |  |  | Residual | 2.921E-005 | 5 | 5.842E-006 |  |  |
| Cor Total | 3296.88 | 15 |  |  |  | Cor Total | 2.054E-003 | 15 |  |  |  |
| **Parameters** | **SS** | **DF** | **MS** | **F** | **p-value** | **Parameters** | **SS** | **DF** | **MS** | **F** | **p-value** |
| **Q8h** |  |  |  |  |  | **Q24** |  |  |  |  |  |
| Model | 938.22 | 10 | 93.82 | 91.68 | < 0.0001 | Model | 41816.17 | 10 | 4181.62 | 24.51 | 0.0013 |
| A | 669.58 | 1 | 669.58 | 654.28 | < 0.0001 | A | 27159.73 | 1 | 27159.73 | 159.17 | < 0.0001 |
| B | 55.62 | 1 | 55.62 | 54.35 | 0.0007 | B | 1318.88 | 1 | 1318.88 | 7.73 | 0.0389 |
| C | 200.48 | 1 | 200.48 | 195.90 | < 0.0001 | C | 10308.14 | 1 | 10308.14 | 60.41 | 0.0006 |
| D | 9.36 | 1 | 9.36 | 9.15 | 0.0293 | D | 2093.46 | 1 | 2093.46 | 12.27 | 0.0172 |
| AB | 0.24 | 1 | 0.24 | 0.23 | 0.6489 | AB | 18.63 | 1 | 18.63 | 0.11 | 0.7545 |
| AC | 1.38 | 1 | 1.38 | 1.35 | 0.2975 | AC | 788.24 | 1 | 788.24 | 4.62 | 0.0843 |
| AD | 9.534E-003 | 1 | 9.534E-003 | 9.316E- 03 | 0.9269 | AD | 28.43 | 1 | 28.43 | 0.17 | 0.7000 |
| BC | 1.52 | 1 | 1.52 | 1.48 | 0.2775 | BC | 30.82 | 1 | 30.82 | 0.18 | 0.6885 |
| BD | 0.023 | 1 | 0.023 | 0.022 | 0.8867 | BD | 54.90 | 1 | 54.90 | 0.32 | 0.5951 |
| CD | 5.096E-003 | 1 | 5.096E-003 | 4.979E-003 | 0.9465 | CD | 14.96 | 1 | 14.96 | 0.088 | 0.7791 |
| Residual | 5.12 | 5 | 1.02 |  |  | Residual | 853.15 | 5 | 170.63 |  |  |
| Cor Total | 943.33 | 15 |  |  |  | Cor Total | 42669.32 | 15 |  |  |  |

**(b)**

Figure S1: *In vitro* release profile of FVS from drug solution and different SNVs formulations: (a) S1-S8 and (b) S9-S16.

**(b)**

Figure S2: Permeation profile of FVS from drug solution and different SNVs formulations: (a) S1–S8 and (b) S9–S16.

Table (S3): *Ex vivo* permeation parameters of FVS-loaded SNVs versus FVS solution

| Formulation | Lag time  (min) | Jss  (µg/cm^2^ h) | Kp  (cm/h) | EI |
| --- | --- | --- | --- | --- |
| S1 | 60.47 ± 10.54 | 9.56 ± 0.85 | 0.0145 ± 0.0008 | 1.79 |
| S2 | 42.58 ± 4.95 | 11.85 ± 0.65 | 0.0195 ± 0.0016 | 2.40 |
| S3 | 56.89 ± 0.57 | 10.65 ± 0.81 | 0.0158 ± 0.0006 | 1.95 |
| S4 | 38.74 ± 6.76 | 12.17 ± 0.88 | 0.0209 ± 0.0026 | 2.58 |
| S5 | 29.87 ± 4.83 | 13.08 ± 0.92 | 0.0237 ± 0.0029 | 2.92 |
| S6 | 21.03 ± 5.35 | 14.56 ± 0.90 | 0.0286 ± 0.0016 | 3.53 |
| S7 | 28.81 ± 5.95 | 13.52 ± 1.06 | 0.0255 ± 0.0014 | 3.15 |
| S8 | 21.67 ± 4.26 | 16.36 ± 0.96 | 0.0323 ± 0.0023 | 3.98 |
| S9 | 50.88 ± 5.86 | 11.09 ± 0.99 | 0.0168 ± 0.0019 | 2.07 |
| S10 | 40.81 ± 0.73 | 12.09 ± 0.83 | 0.0203 ± 0.0025 | 2.51 |
| S11 | 42.53 ± 7.00 | 11.56 ± 0.94 | 0.0177 ± 0.0021 | 2.19 |
| S12 | 36.25 ± 5.21 | 12.87 ± 0.88 | 0.0216 ± 0.0027 | 2.67 |
| S13 | 31.17 ± 3.35 | 13.54 ± 1.04 | 0.0277 ± 0.0028 | 3.42 |
| S14 | 18.61 ± 2.71 | 16.77 ± 1.06 | 0.0332 ± 0.0012 | 4.09 |
| S15 | 24.53 ± 5.90 | 14.10 ± 0.90 | 0.0283 ± 0.0026 | 3.49 |
| S16 | 16.99 ± 1.17 | 19.18 ± 0.83 | 0.0362 ± 0.0017 | 4.46 |
| Drug solution | 85.32 ± 14.03 | 8.81 ± 0.48 | 0.0081 ± 0.0016 | -- |

Jss: drug flux; Kp: permeability coefficient; EI: enhancement index.

Data are mean values (n = 3) ± SD.

Table (S4): Composition, actual and predicted responses of the optimal FVS-loaded SNVs formulation

| **Factor** | **Optimal value** | **Response**  **Variable** | **Actual**  **value** | **Predicted value** | % **Prediction error^b^** |
| --- | --- | --- | --- | --- | --- |
| A:Span type | Span 80 | EE% | 71.28 | 72.34 | - 1.49 |
| B:EA type | Brij 35 | Vesicle size  (nm) | 201.54 | 210.73 | - 4.56 |
| C:EA concentration (% w/w)^a^ | 20 | Q_8h_  (%) | 89.45 | 91.07 | - 1.81 |
| Sonication time  (min) | 0 | Q_24_  (µg/cm^2^) | 402.55 | 389.95 | 3.13 |

EA: edge activator; EE%: entrapment efficiency percent; Q_8h_: cumulative release after 8 h; Q_24_: cumulative amount permeated/unit area in 24 h.

^a^ (% w/w) of the EA with respect to Span/EA total weight.

^b^Calculated as [Actual-predicted/Acctual] *100.


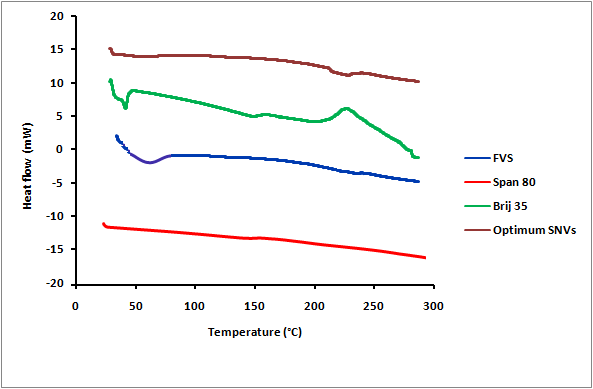

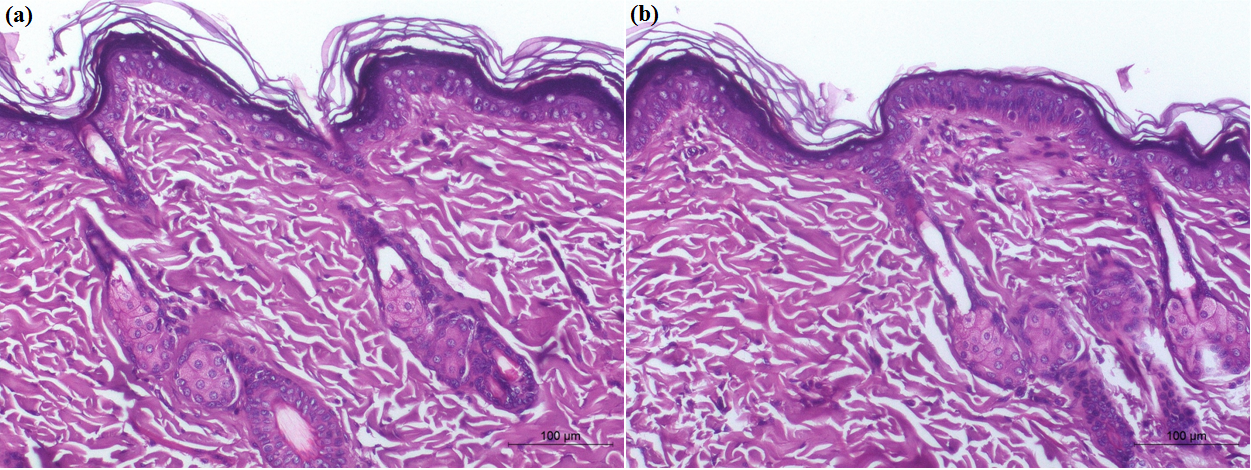
Figure S3: DSC thermograms of FVS, Span 80, Brij 35 and the optimum SNVs.

Figure S4: Light photomicrographs showing histopathological sections of (a) normal untreated rat skin and (b) rat skin treated with FVS-loaded SNVs gel (X200 H&E stain).


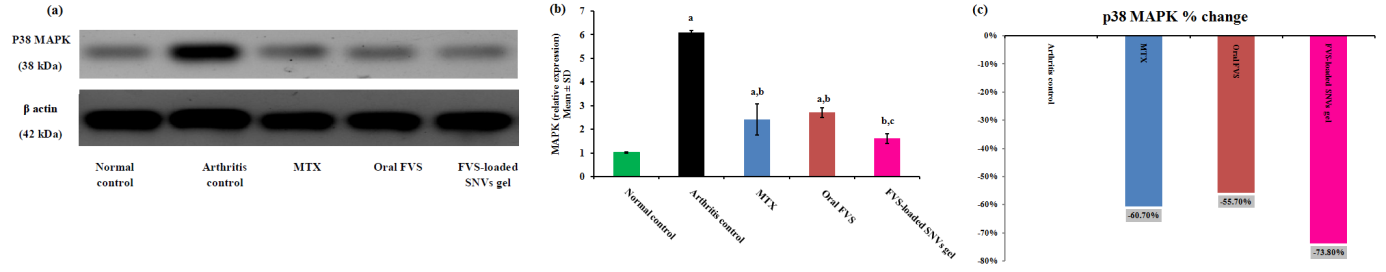
Figure S5: Effect of transdermal application of FVS-loaded SNVs gel on p38 MAPK protein expression and its % change as compared to arthritis control group using western blot analysis. Three replicates were used for each group.

^a^Significantly different from normal control group value at *p* < 0.05.

^b^Significantly different from arthritis control group value at *p* < 0.05.

^c^Significantly different from oral FVS-treated group value at *p* < 0.05.

p38 MAPK: p38 mitogen-activated protein kinase; MTX: methotrexate; FVS: fluvastatin; SNVs: spanlastic nanovesicles.

Table (S5): Storage stability of the optimized SNVs formulation

| Time  (months) | EE  (%) | Particle size  (nm) | PDI |
| --- | --- | --- | --- |
| 0 | 71.28 ± 2.05 | 201.54 ± 9.16 | 0.31 |
| 1 | 69.72 ± 2.83 | 205.43 ± 10.23 | 0.34 |
| 2 | 69.12 ± 1.49 | 211.76 ± 9.85 | 0.39 |
| 3 | 68.89 ± 1.77 | 216.65 ± 11.64 | 0.43 |

EE, entrapment efficiency; PDI, polydispersity index.

Data represent mean ± SD (n = 3), *p* ˂ 0.05 for one way ANOVA followed by Dunnett multiple comparison test.
